# (3a*R*,8a*S*,9*S*,9a*R*)-9-Hydroxy­perhydro­furo[3,2-*f*]indolizin-6-one

**DOI:** 10.1107/S1600536809024283

**Published:** 2009-07-01

**Authors:** Ľubomír Švorc, Viktor Vrábel, Jozefína Žúžiová, Štefan Marchalín, Jozef Kožíšek

**Affiliations:** a Institute of Analytical Chemistry, Faculty of Chemical and Food Technology, Slovak Technical University, Radlinského 9, SK-812 37 Bratislava, Slovak Republic 81237; b Institute of Organic Chemistry, Catalysis and Petrochemistry, Faculty of Chemical and Food Technology, Slovak Technical University, Radlinského 9, SK-812 37 Bratislava, Slovak Republic 81237; c Institute of Physical Chemistry and Chemical Physics, Faculty of Chemical and Food Technology, Slovak Technical University, Radlinského 9, Bratislava, Slovak Republic 81237

## Abstract

In the title compound, C_10_H_15_NO_3_, the central six-membered ring of the indolizine system adopts a chair conformation, while the oxopyrrolidine and hydro­furan rings attached to the indolizine ring system have envelope conformations. In the crystal, the mol­ecules form chains parallel to the *b* axis *via* inter­molecular O—H⋯O hydrogen bonds. The absolute configuration was assigned from the synthesis.

## Related literature

For general properties of indolizines see: Gundersen *et al.* (2007 ▶); Sundaram *et al.* (2007 ▶); Mikael (1999 ▶); Pyne (2005 ▶); Karanjule *et al.* (2006 ▶); Chaudhari *et al.* (2006 ▶); Martin *et al.* (2005 ▶). For the synthesis of the title compound see: Šafář *et al.* (2008 ▶). For related structures, see: Vrábel *et al.* (2004 ▶); Švorc *et al.* (2009 ▶). Camus *et al.* (2003 ▶) For puckering parameters, see: Cremer & Pople (1975 ▶).
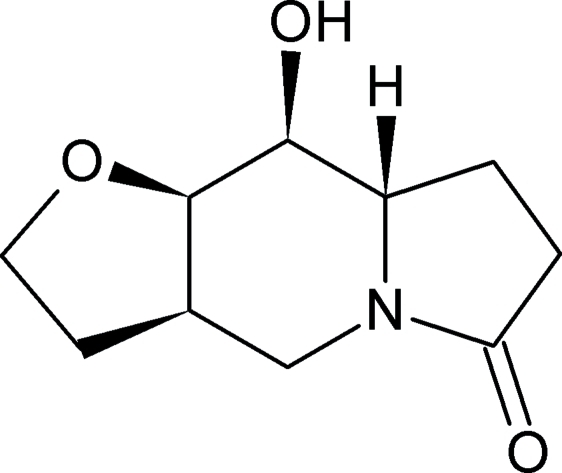


## Experimental

### 

#### Crystal data

C_10_H_15_NO_3_
*M* *_r_* = 197.23
Monoclinic, 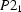

*a* = 6.2856 (1) Å
*b* = 6.4521 (1) Å
*c* = 11.7698 (2) Å
β = 98.631 (2)°
*V* = 471.92 (1) Å^3^
*Z* = 2
Mo *K*α radiation
μ = 0.10 mm^−1^
*T* = 298 K
0.45 × 0.29 × 0.04 mm

#### Data collection

Oxford Diffraction Gemini R CCD diffractometer
Absorption correction: analytical (Clark & Reid, 1995 ▶) *T*_min_ = 0.962, *T*_max_ = 0.996
12197 measured reflections
1359 independent reflections
1151 reflections with *I* > 2σ(*I*)
*R*_int_ = 0.024

#### Refinement

*R*[*F* ^2^ > 2σ(*F* ^2^)] = 0.032
*wR*(*F* ^2^) = 0.088
*S* = 1.09
1359 reflections
131 parameters
1 restraint
H atoms treated by a mixture of independent and constrained refinement
Δρ_max_ = 0.18 e Å^−3^
Δρ_min_ = −0.12 e Å^−3^

### 

Data collection: *CrysAlis CCD* (Oxford Diffraction, 2006 ▶); cell refinement: *CrysAlis RED* (Oxford Diffraction, 2006 ▶); data reduction: *CrysAlis RED*; program(s) used to solve structure: *SHELXS97* (Sheldrick, 2008 ▶); program(s) used to refine structure: *SHELXL97* (Sheldrick, 2008 ▶); molecular graphics: *DIAMOND* (Brandenburg, 2001 ▶); software used to prepare material for publication: *enCIFer* (Allen *et al.*, 2004 ▶).

## Supplementary Material

Crystal structure: contains datablocks I, global. DOI: 10.1107/S1600536809024283/bg2263sup1.cif

Structure factors: contains datablocks I. DOI: 10.1107/S1600536809024283/bg2263Isup2.hkl

Additional supplementary materials:  crystallographic information; 3D view; checkCIF report

## Figures and Tables

**Table 1:** Hydrogen-bond geometry (Å, °)

*D*—H⋯*A*	*D*—H	H⋯*A*	*D*⋯*A*	*D*—H⋯*A*
O2—H2*A*⋯O3^i^	0.82 (3)	2.15 (3)	2.9233 (19)	157 (2)

Symmetry code: (i) 
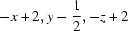
.

**Fig. 1.**
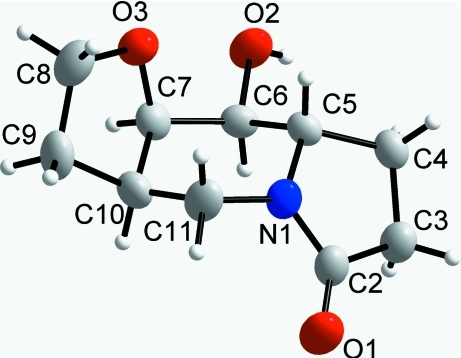
Molecular structure of (I) with the atomic numbering scheme. Displacement ellipsoids are drawn at the 50% probability level (Brandenburg, 2001).

**Fig. 2.**
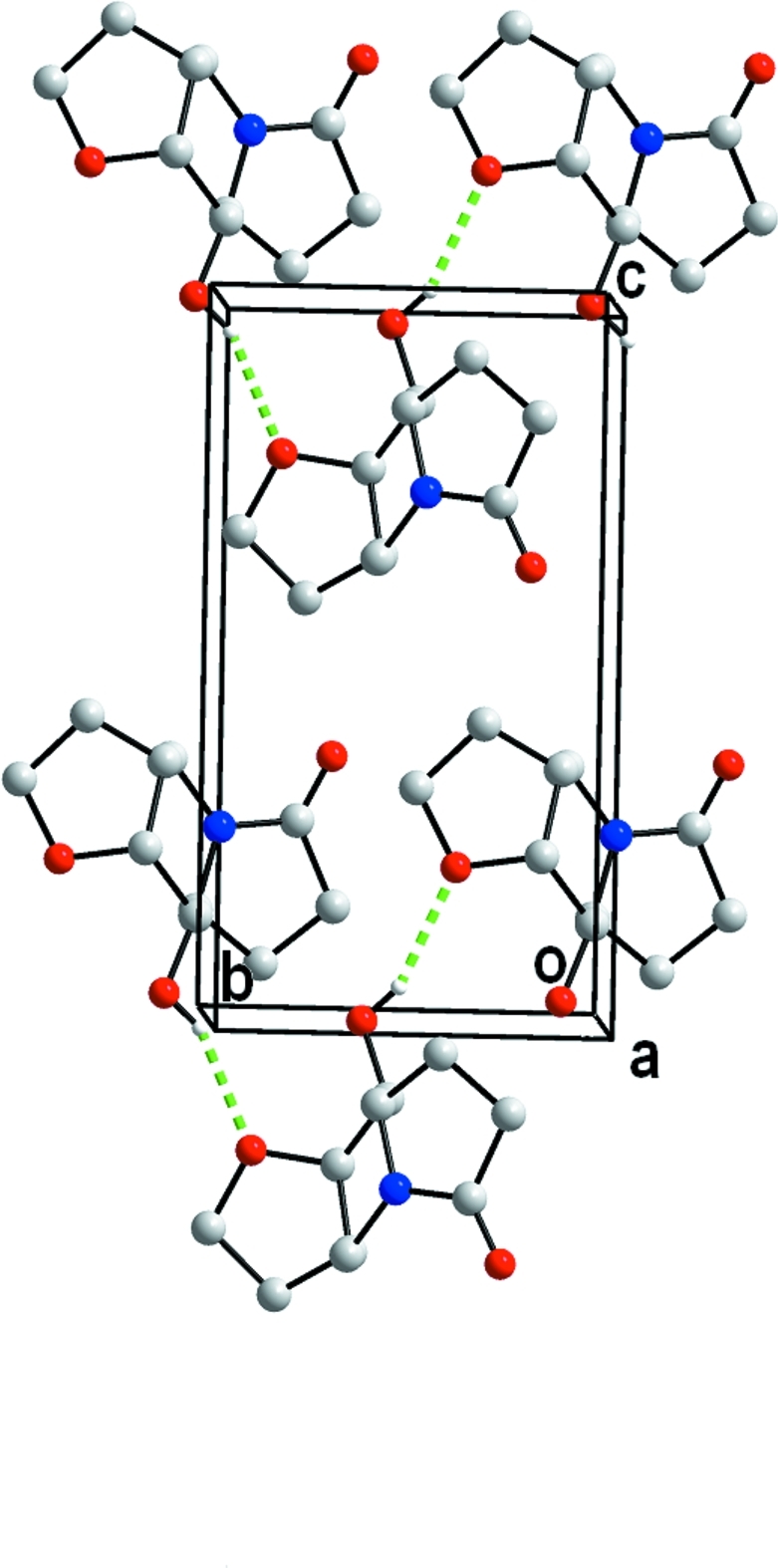
Packing view of (I), projected along a and showing the formation of chains running along b.

## supplementary crystallographic information

### Comment

Indolizines are electron-rich heterocycles with very low oxidation potential. Functionalized indolizines are common substructures found in biologically important natural products and synthetic pharmaceuticals. Due to the various biological functions associated with this skeleton, it has been frequently employed as a key scaffold in the drug industry (Gundersen *et al.*, 2007). The indolizine derivatives show antibacterial, antiviral, antiherpes, anticancer, antifungal, antihelmintic and insecticidal activity (Sundaram *et al.*, 2007). Indolizine alkaloids are excellent inhibitors of biologically important pathways. These include the binding and processing of glycoproteins, potent glycosidase inhibitory activities (Pyne, 2005), activity against AIDS virus HIV and some carcinogenic cells (Mikael, 1999). Castanospermine (Karanjule *et al.*, 2006), swainsonine (Martin *et al.*, 2005) and lentiginosine (Chaudhari *et al.*, 2006) have shown respective glycosidase and mannosidase inhibitory activities, respectively. While an impressive number of total syntheses of polyhydroxylated indolizines and their non-natural analogues in chiral or racemic forms have been reported, the monohydroxylated indolizines have attracted far less attention.

Based on these facts and in contitutation of our interest in developing simple and efficient route for the synthesis of novel monohydroxylated indolizine derivatives, we report here the synthesis, molecular and crystal structure of the title compound, (I). The absolute configuration was established by synthesis and is depicted in the scheme and figure. The expected stereochemistry of atoms C5, C6, C7 and C10 was confirmed as S, S, *R* and *R*, respectively (Fig. 1). The central six-membered ring is not planar and adopts a chair conformation (Cremer & Pople, 1975). A calculation of least-squares planes shows that this ring is puckered in such a manner that the four atoms C5, C6, C10 and C11 are coplanar to within 0.019 (2) Å, while atoms N1 and C7 are displaced from this plane on opposite sides, with out-of-plane displacements of -0.591 (2) and 0.565 (1) Å, respectively. The oxopyrrolidine and hydrofuran rings are each distorted towards an envelope conformation, with atoms C4 and C10 as the flaps. The displacements of atoms C4 and C10 from the mean planes of the remaining four atoms are 0.316 (2) and 0.642 (2) Å, respectively. The central six-membered N-ring is approximately perpendicular to the hydrofuran ring (dihedral angle between plane defined by atoms C5, C6, C10 and C11 and plane defined by atoms C7, O3, C8 and C9) is 82.2 (1)°. As was mentioned in previous papers (Vrábel *et al.*, 2004; Švorc *et al.*, 2009), the N1—C5 and N1—C11 bonds are approximately equivalent and both are much longer than the N1—C2 bond. Atom N1 is *sp*^2^-hybridized, as evidenced by the sum of the valence angles around it (357.3 (2)°). These data are consistent with conjugation of the lone-pair electrons on N1 with the adjacent carbonyl, similar to what is observed for amides. Intermolecular O—H···O hydrogen bonds link the molecules of (I) into extended chains, which run parallel to the *b* axis (Fig. 2) and help to stabilize the crystal structure of the compound. Atom O2 participates as acceptor and atom O3 as donator in these intermolecular hydrogen bonds. Bond lengths and angles in the indolizine ring system are in good agreement with values from the literature (Camus *et al.*, 2003).

### Experimental

The title compound (3aR,8aS,9*S*,9aR)-9-hydroxyoctahydrofuro[3,2-*f*]indolizin- 6(4*H*)-one was prepared according literature procedures of Šafář *et al.* (2008).

### Refinement

Atom H2 was refined isotropically. All other H atoms were positioned geometrically and treated as riding atoms, with C—H distances in the range 0.97 - 0.98Å and *U*_iso_ set at 1.2*U*_eq_ of the parent atom. The absolute configuration could not be reliably determined for this compound using Mo radiation, and has been assigned according to the synthesis; Friedel pairs have been merged.

### Crystal data

**Table:**

C_10_H_15_NO_3_	*F*(000) = 212
*M**_r_* = 197.23	*D*_x_ = 1.388 Mg m^−^^3^
Monoclinic, *P*2_1_	Mo *K*α radiation, λ = 0.71073 Å
Hall symbol: P 2yb	Cell parameters from 7268 reflections
*a* = 6.2856 (1) Å	θ = 3.2–29.4°
*b* = 6.4521 (1) Å	µ = 0.10 mm^−^^1^
*c* = 11.7698 (2) Å	*T* = 298 K
β = 98.631 (2)°	Block, white
*V* = 471.92 (1) Å^3^	0.45 × 0.29 × 0.04 mm
*Z* = 2	

### Data collection

**Table:**

Oxford Diffraction Gemini R CCD diffractometer	1359 independent reflections
Radiation source: fine-focus sealed tube	1151 reflections with *I* > 2σ(*I*)
graphite	*R*_int_ = 0.024
Detector resolution: 10.4340 pixels mm^-1^	θ_max_ = 29.5°, θ_min_ = 3.5°
Rotation method data acquisition using ω and φ scans	*h* = −8→8
Absorption correction: analytical (Clark & Reid, 1995)	*k* = −8→8
*T*_min_ = 0.962, *T*_max_ = 0.996	*l* = −16→16
12197 measured reflections	

### Refinement

**Table:**

Refinement on *F*^2^	Primary atom site location: structure-invariant direct methods
Least-squares matrix: full	Secondary atom site location: difference Fourier map
*R*[*F*^2^ > 2σ(*F*^2^)] = 0.032	Hydrogen site location: inferred from neighbouring sites
*wR*(*F*^2^) = 0.088	H atoms treated by a mixture of independent and constrained refinement
*S* = 1.09	*w* = 1/[σ^2^(*F*_o_^2^) + (0.0589*P*)^2^ + 0.002*P*] where *P* = (*F*_o_^2^ + 2*F*_c_^2^)/3
1359 reflections	(Δ/σ)_max_ < 0.001
131 parameters	Δρ_max_ = 0.18 e Å^−^^3^
1 restraint	Δρ_min_ = −0.12 e Å^−^^3^

### Special details

**Table:**

Experimental. (face-indexed; Oxford Diffraction, 2006)
Geometry. All e.s.d.'s (except the e.s.d. in the dihedral angle between two l.s. planes) are estimated using the full covariance matrix. The cell e.s.d.'s are taken into account individually in the estimation of e.s.d.'s in distances, angles and torsion angles; correlations between e.s.d.'s in cell parameters are only used when they are defined by crystal symmetry. An approximate (isotropic) treatment of cell e.s.d.'s is used for estimating e.s.d.'s involving l.s. planes.
Refinement. Refinement of *F*^2^ against ALL reflections. The weighted *R*-factor *wR* and goodness of fit *S* are based on *F*^2^, conventional *R*-factors *R* are based on *F*, with *F* set to zero for negative *F*^2^. The threshold expression of *F*^2^ > σ(*F*^2^) is used only for calculating *R*-factors(gt) *etc*. and is not relevant to the choice of reflections for refinement. *R*-factors based on *F*^2^ are statistically about twice as large as those based on *F*, and *R*- factors based on ALL data will be even larger.

### Fractional atomic coordinates and isotropic or equivalent isotropic displacement parameters (Å^2^)

**Table:**

	*x*	*y*	*z*	*U*_iso_*/*U*_eq_	
C2	0.4873 (3)	0.2817 (3)	0.72325 (14)	0.0411 (4)	
C3	0.4877 (3)	0.1873 (3)	0.84157 (15)	0.0459 (4)	
H3B	0.3467	0.1332	0.8494	0.055*	
H3A	0.5921	0.0759	0.8550	0.055*	
C4	0.5484 (3)	0.3645 (3)	0.92481 (13)	0.0387 (4)	
H4B	0.6470	0.3177	0.9912	0.046*	
H4A	0.4216	0.4225	0.9506	0.046*	
C5	0.6564 (2)	0.5248 (3)	0.85636 (11)	0.0332 (3)	
H5A	0.6105	0.6647	0.8742	0.040*	
C6	0.9007 (2)	0.5135 (3)	0.87248 (12)	0.0344 (3)	
H6A	0.9426	0.3684	0.8650	0.041*	
C7	0.9925 (2)	0.6393 (3)	0.78316 (14)	0.0380 (4)	
H7A	1.1473	0.6117	0.7900	0.046*	
C8	0.9599 (3)	0.9602 (3)	0.69052 (17)	0.0550 (5)	
H8B	1.0849	1.0494	0.6934	0.066*	
H8A	0.8315	1.0444	0.6723	0.066*	
C9	0.9646 (3)	0.7923 (4)	0.60051 (16)	0.0532 (5)	
H9B	1.1088	0.7732	0.5823	0.064*	
H9A	0.8680	0.8251	0.5306	0.064*	
C10	0.8887 (3)	0.5997 (3)	0.65890 (13)	0.0431 (4)	
H10A	0.9444	0.4726	0.6286	0.052*	
C11	0.6434 (3)	0.5962 (3)	0.64646 (13)	0.0468 (4)	
H11B	0.5898	0.7367	0.6500	0.056*	
H11A	0.5847	0.5383	0.5723	0.056*	
N1	0.5732 (2)	0.4730 (2)	0.73730 (11)	0.0396 (3)	
O1	0.4241 (2)	0.1993 (3)	0.63124 (11)	0.0624 (4)	
O2	0.9924 (2)	0.5850 (2)	0.98351 (11)	0.0487 (3)	
H2A	0.993 (4)	0.493 (5)	1.031 (2)	0.060 (7)*	
O3	0.9608 (2)	0.85665 (19)	0.79966 (10)	0.0471 (3)	

### Atomic displacement parameters (Å^2^)

**Table:**

	*U*^11^	*U*^22^	*U*^33^	*U*^12^	*U*^13^	*U*^23^
C2	0.0366 (7)	0.0539 (10)	0.0319 (7)	−0.0097 (7)	0.0025 (6)	0.0024 (7)
C3	0.0520 (9)	0.0514 (10)	0.0339 (8)	−0.0129 (8)	0.0044 (7)	0.0047 (7)
C4	0.0391 (7)	0.0485 (10)	0.0291 (7)	−0.0010 (7)	0.0071 (6)	0.0031 (7)
C5	0.0373 (7)	0.0380 (8)	0.0242 (6)	0.0012 (6)	0.0045 (5)	−0.0003 (6)
C6	0.0375 (7)	0.0378 (8)	0.0263 (7)	0.0009 (6)	−0.0004 (5)	−0.0011 (7)
C7	0.0325 (7)	0.0471 (10)	0.0345 (8)	0.0000 (7)	0.0057 (6)	0.0001 (7)
C8	0.0631 (11)	0.0518 (11)	0.0482 (10)	−0.0164 (9)	0.0025 (8)	0.0130 (9)
C9	0.0532 (9)	0.0700 (13)	0.0378 (9)	−0.0141 (10)	0.0116 (7)	0.0122 (9)
C10	0.0545 (9)	0.0477 (10)	0.0290 (7)	−0.0050 (8)	0.0129 (6)	−0.0009 (7)
C11	0.0544 (9)	0.0563 (10)	0.0271 (7)	−0.0143 (9)	−0.0026 (6)	0.0087 (8)
N1	0.0407 (7)	0.0506 (8)	0.0255 (6)	−0.0095 (6)	−0.0019 (5)	0.0053 (6)
O1	0.0721 (8)	0.0765 (10)	0.0357 (6)	−0.0308 (8)	−0.0011 (6)	−0.0067 (7)
O2	0.0583 (7)	0.0559 (8)	0.0275 (6)	−0.0166 (6)	−0.0082 (5)	0.0040 (6)
O3	0.0619 (7)	0.0427 (7)	0.0362 (6)	−0.0111 (6)	0.0053 (5)	0.0005 (6)

### Geometric parameters (Å, °)

**Table:**

C2—O1	1.218 (2)	C7—C10	1.531 (2)
C2—N1	1.347 (2)	C7—H7A	0.9800
C2—C3	1.520 (2)	C8—O3	1.447 (2)
C3—C4	1.517 (3)	C8—C9	1.519 (3)
C3—H3B	0.9700	C8—H8B	0.9700
C3—H3A	0.9700	C8—H8A	0.9700
C4—C5	1.530 (2)	C9—C10	1.531 (3)
C4—H4B	0.9700	C9—H9B	0.9700
C4—H4A	0.9700	C9—H9A	0.9700
C5—N1	1.4591 (18)	C10—C11	1.527 (2)
C5—C6	1.5204 (19)	C10—H10A	0.9800
C5—H5A	0.9800	C11—N1	1.453 (2)
C6—O2	1.4238 (17)	C11—H11B	0.9700
C6—C7	1.510 (2)	C11—H11A	0.9700
C6—H6A	0.9800	O2—H2A	0.82 (3)
C7—O3	1.433 (2)		
			
O1—C2—N1	125.45 (17)	C6—C7—H7A	108.9
O1—C2—C3	126.54 (18)	C10—C7—H7A	108.9
N1—C2—C3	108.00 (15)	O3—C8—C9	106.96 (17)
C4—C3—C2	104.81 (15)	O3—C8—H8B	110.3
C4—C3—H3B	110.8	C9—C8—H8B	110.3
C2—C3—H3B	110.8	O3—C8—H8A	110.3
C4—C3—H3A	110.8	C9—C8—H8A	110.3
C2—C3—H3A	110.8	H8B—C8—H8A	108.6
H3B—C3—H3A	108.9	C8—C9—C10	103.06 (14)
C3—C4—C5	104.93 (12)	C8—C9—H9B	111.2
C3—C4—H4B	110.8	C10—C9—H9B	111.2
C5—C4—H4B	110.8	C8—C9—H9A	111.2
C3—C4—H4A	110.8	C10—C9—H9A	111.2
C5—C4—H4A	110.8	H9B—C9—H9A	109.1
H4B—C4—H4A	108.8	C11—C10—C9	110.32 (16)
N1—C5—C6	108.57 (12)	C11—C10—C7	111.95 (13)
N1—C5—C4	103.15 (13)	C9—C10—C7	100.16 (14)
C6—C5—C4	114.91 (13)	C11—C10—H10A	111.3
N1—C5—H5A	110.0	C9—C10—H10A	111.3
C6—C5—H5A	110.0	C7—C10—H10A	111.3
C4—C5—H5A	110.0	N1—C11—C10	110.56 (13)
O2—C6—C7	108.67 (13)	N1—C11—H11B	109.5
O2—C6—C5	111.18 (13)	C10—C11—H11B	109.5
C7—C6—C5	111.84 (12)	N1—C11—H11A	109.5
O2—C6—H6A	108.4	C10—C11—H11A	109.5
C7—C6—H6A	108.4	H11B—C11—H11A	108.1
C5—C6—H6A	108.4	C2—N1—C11	124.84 (15)
O3—C7—C6	110.87 (13)	C2—N1—C5	114.01 (14)
O3—C7—C10	104.17 (14)	C11—N1—C5	118.47 (13)
C6—C7—C10	114.99 (13)	C6—O2—H2A	110.8 (18)
O3—C7—H7A	108.9	C7—O3—C8	108.27 (13)
			
O1—C2—C3—C4	−171.33 (18)	O3—C7—C10—C9	41.27 (16)
N1—C2—C3—C4	9.79 (19)	C6—C7—C10—C9	162.80 (14)
C2—C3—C4—C5	−19.80 (18)	C9—C10—C11—N1	−156.02 (15)
C3—C4—C5—N1	22.25 (17)	C7—C10—C11—N1	−45.4 (2)
C3—C4—C5—C6	−95.73 (16)	O1—C2—N1—C11	−12.7 (3)
N1—C5—C6—O2	173.44 (14)	C3—C2—N1—C11	166.20 (16)
C4—C5—C6—O2	−71.68 (18)	O1—C2—N1—C5	−173.78 (17)
N1—C5—C6—C7	51.76 (17)	C3—C2—N1—C5	5.11 (19)
C4—C5—C6—C7	166.64 (13)	C10—C11—N1—C2	−105.48 (18)
O2—C6—C7—O3	−54.82 (17)	C10—C11—N1—C5	54.8 (2)
C5—C6—C7—O3	68.30 (16)	C6—C5—N1—C2	104.77 (16)
O2—C6—C7—C10	−172.63 (14)	C4—C5—N1—C2	−17.57 (17)
C5—C6—C7—C10	−49.52 (19)	C6—C5—N1—C11	−57.6 (2)
O3—C8—C9—C10	19.3 (2)	C4—C5—N1—C11	−179.96 (15)
C8—C9—C10—C11	82.07 (18)	C6—C7—O3—C8	−154.92 (13)
C8—C9—C10—C7	−36.05 (18)	C10—C7—O3—C8	−30.69 (17)
O3—C7—C10—C11	−75.64 (18)	C9—C8—O3—C7	7.04 (19)
C6—C7—C10—C11	45.9 (2)		

### Hydrogen-bond geometry (Å, °)

**Table:**

*D*—H···*A*	*D*—H	H···*A*	*D*···*A*	*D*—H···*A*
O2—H2A···O3^i^	0.82 (3)	2.15 (3)	2.9233 (19)	157 (2)

Symmetry codes: (i) −*x*+2, *y*−1/2, −*z*+2.
